# Inhibition of choroidal neovascularization by homoisoflavanone, a new angiogenesis inhibitor

**Published:** 2008-03-18

**Authors:** Jeong Hun Kim, Jin Hyoung Kim, Young Suk Yu, Hyoung-Oh Jun, Ho Jeong Kwon, Kyu Hyung Park, Kyu-Won Kim

**Affiliations:** 1 Department of Ophthalmology, College of Medicine, Seoul National University and Seoul Artificial Eye Center Clinical Research Institute, Seoul National University Hospital, Seoul, Korea;; 2 Neurovascular Coordination Research Center, College of Pharmacy and Research Institute of Pharmaceutical Sciences, Seoul National University, Seoul, Korea;; 3 Chemical Genomics Laboratory, Department of Biotechnology, College of Engineering, Yonsei University, Seoul, Korea;; 4 Department of Ophthalmology, Bundang Seoul National University Hospital, Seoul, Korea

## Abstract

**Purpose:**

Age-related macular degeneration (AMD) is the leading cause of blindness in elderly. The detailed mechanism of choroidal neovascularization (CNV) leads to severe vision loss in patients with AMD. This study was undertaken to evaluate the inhibitory effect of homoisoflavanone on CNV.

**Methods:**

Antiangiogenic activity of homoisoflavanone was evaluated by in vitro tube formation assay of human umbilical vein endothelial cells (HUVECs) and cell migration assay of HUVECs., Homoisoflavanone or PBS was injected intravitreously into a mouse model of laser-photocoagulation-induced CNV. Fluorescein angiography and vessel counting in cross sections were employed to examine CNV lesions. The toxicity of homoisoflavanone was evaluated through 3-(4,5-Dimethylthiazol-2-yl)-2,5-diphenyltetrazolium bromide assay (MTT) assay in HUVECs as well as histological examination and terminal deoxynucleotidyl transferase biotin-dUTP nick end labeling (TUNEL) staining in the retina.

**Results:**

Homoisoflavanone effectively inhibited in vitro tube formation and cell migration of HUVECs. Interestingly, homoisoflavanone significantly reduced CNV and its leakage in a mouse model of laser-photocoagulation-induced CNV. In addition, homoisoflavanone showed no effect on cell viability of HUVECs and no retinal toxicity in a concentration range of 1-10 μM.

**Conclusions:**

Our data suggest that homoisoflavanone is a potent inhibitor of CNV and may be applied in the treatment of other vasoproliferative retinopathies and tumor.

## Introduction

Angiogenesis is a process that forms new blood vessels; it is tightly regulated by a balance between positive and negative factors [1]. Normally, it does not occur except during developmental or vessel repair. Pathological angiogenesis in the eye is the most common cause of blindness in all age groups. For example, retinopathy of prematurity (ROP) is the common cause for children, diabetic retinopathy for young adults, and age-related macular degeneration for elderly [2].

In developed countries, AMD is the leading cause of blindness in adults 55 years and older [3]. Choroidal neovascularization (CNV) can lead to severe vision loss in patients with AMD [4]. CNV occurs through vessel proliferation from the choroidal vessels invading the subretinal space after the rupture of Bruch’s membrane followed by the vessel proliferation from the choroidal vessels invading the subretinal space. CNV vessels are fragile and leaky, leading to hemorrhage or exudation, which injures photoreceptor cells. Further, these proliferative vessels induce the formation of fibrovascular scarring, which results in irreversible damage to retinal function and can lead to blindness [5]. Since excessive proliferation of vascular cells is the essential mechanism of CNV, a reasonable therapeutic approach is to directly suppress the proliferating vascular endothelial cells. 

The homoisoflavonone, 5,7-dihydroxy-3-(3-hydroxy-4- methoxybenzyl)-6-methoxychroman-4-1, was found in the bulb of Cremastra appendiculata which has been traditionally known as a medicinal plant in East Asia. We determined that homoisoflavanone is a potent angiogenesis inhibitor in the course of our research for new angiogenesis inhibitors from natural products [7]. Moreover, we showed that homoisoflavanone inhibits retinal neovascularization, which is related to G_2_/M phase cell cycle arrest with increase of p21^WAF1^ expression [8]. 

The most common model of CNV is created by laser-photocoagulation-induced rupture of Bruch’s membrane, which stimulates neovascularization from preexisting choroidal capillary networks [6]. In the present study, we demonstrate homoisoflavanone inhibits in vitro angiogenesis of human umbilical vein endothelial cells (HUVECs) without cytotoxic effect under therapeutic concentration range of 1-10 μM, and significantly reduces CNV in a laser CNV model without significant retinal toxicity on the therapeutic concentration. Herein, we suggest that homoisoflavanone may have therapeutic potential in the treatment of CNV of AMD as well as in other vasoproliferative retinopathies, such as retinopathy of prematurity and diabetic retinopathy, and tumors.

## Methods

### Animals

Female C57BL/6 mice, aged 7- 8 weeks, were purchased from Samtako (Gyeonggi-do, Korea). Care, use, and treatment of all animals in this study were in strict agreement with the ARVO statement for the Use of Animals in Ophthalmic and Vision Research.

### Preparation of homoisoflavanone

Homoisoflavanone was extracted from the bulb of Cremastra appendiculata (Seoul, Korea) by eluting with ethanol as previously described [7]. The final product was more than 99% pure. Homoisoflavanone was stored at a stock concentration of 1mM in a nitrogen tank.

**Figure 1:**
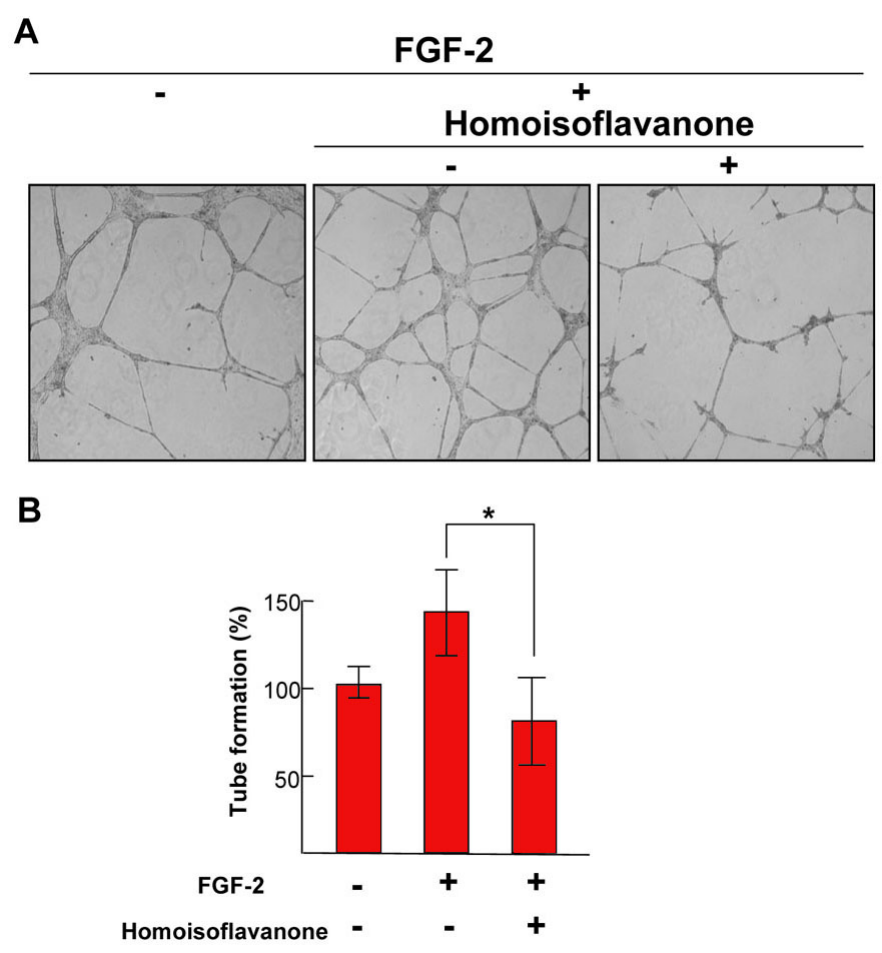
Effect of homoisoflavanone on fibroblast growth factor-2 and human umbilical vein endothelial cells. **A:** Each figure is representative of three independent experiments. **B:** Basal tube formation of human umbilical vein endothelial cells (HUVECs) left in serum-free media was normalized to 100% respectively. Each value represents means ± SEM from three independent experiments (*p<0.05).

### Tube formation assay

Tube formation was assayed as described previously [9]. The air-dried bulb was milled and extracted in 85% ethanol, which was filtered and concentrated under vacuum. The ethanol extract was separated by silica gel column chromatography. HUVECs (1×10^5^ cells) were inoculated on the surface of the Matrigel (Sigma–Aldrich Ltd., St. Louis, MO), and treated with 1 μM homoisoflavanone or fibroblast growth factor (FGF)-2 for 18 h. The morphological changes of the cells and tubes formed were observed under a microscope (Carl Zeiss, Chester, VA) and photographed at a ×200 magnification. Tube formation was quantified by counting the number of connected cells in randomly selected fields at a ×200 magnification with a microscope, and dividing that number by the total number of cells in the same field.

### Endothelial cell migration assay

Chemotactic motility of HUVECs was assayed as described previously [9]. Briefly, the lower surface of the filter was coated with gelatin. One hundred microliters of the cell suspension was loaded into each of the upper wells, and the chamber was incubated at 37 °C for 4 h. The cells were fixed and stained with hematoxylin and eosin. Nonmigrating cells on the upper surface of the filter were removed by wiping with a cotton swab. The migration assay of endothelial cells was performed in vitro using a Transwell chamber system with 8.0 μm pore-sized polycarbonate filter inserts (Corning Costar, Cambridge, MA). The total number of migrated cells in the lower side of the filter was quantified at a ×200 magnification with a microscope(Carl Zeiss, Chester, VA). Ten fields were counted for each assay.

**Figure 2:**
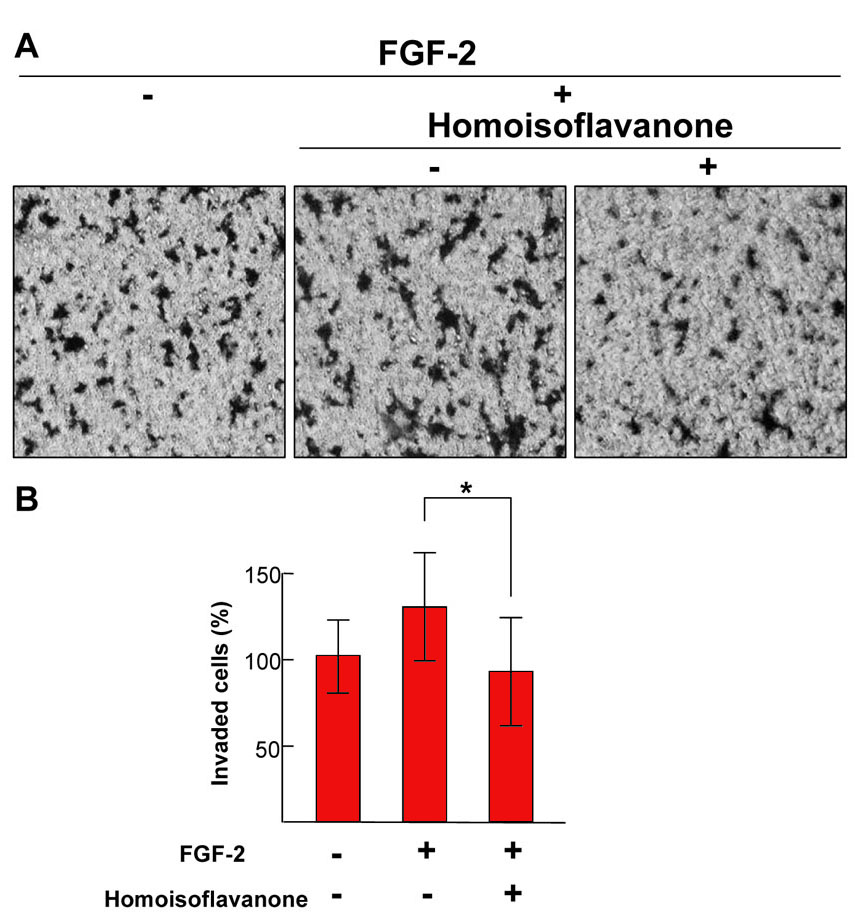
Effect of homoisoflavanone on fibroblast growth factor-2-induced cell migration of human umbilical vein endothelial cells. **A:** Each figure is representative of three independent experiments. **B:** The basal migration of human umbilical vein endothelial cells (HUVECs) that were left in serum-free media was normalized to 100% respectively. Each value represents means ± SEM from three independent experiments (*p<0.05).

### Cell viability assay

Cell viability was evaluated with the 3-(4,5-dimethylthiazol-2-yl)-2,5-diphenyltetrazolium bromide (MTT) assay. HUVECs (1x10^5^ cells) were plated in 96-well plates and cultured overnight. Cells were treated with 1-10 μM homoisoflavanone for 48 h. The medium was then replaced with fresh medium containing 0.5 mg/mL MTT for 4 h. After incubation, the medium was carefully removed from the plate, and Dimethyl sulfoxide (DMSO) was added to solubilize formazan produced from MTT by the viable cells. Absorbance was measured at 540 nm using a microplate reader (Molecular Devices, Sunnyvale, CA).

### Laser-photocoagulation-induced choroidal neovascularization

Female C57BL/6J mice, aged 7-8 weeks, were anesthesized with an intraperitoneal injection of ketamine-xylazine (10 mg/kg), and the pupils were dilated with 1% tropicamide (Alcon Laboratories Inc., Fort Worth, TX). The burn of a 831-nm 106 diode laser photocoagulation (75 μm spot size, 0.1 s duration, 120 mW) was delivered to each 3, 6, 9, and 12 o’clock position of two disc diameters from optic disc by using an indirect head set delivery system of a photocoagulator (OcuLight; Iridex, Mountain View, CA) and a handheld +78 diopter lens. The bubbling or pop sensing with laser photocoagulation was considered as the successful rupture of Bruch’s membrane. The cases of successful rupture were included in this study.

To assess the antiangiogenic activity of homoisoflavanone, we injected the mice intravitreously with 1 µM homoisoflavanone in 1 λ PBS 10 days after laser photocoagulation, when maximum CNV began. These experiments were repeated 27 times.

**Figure 3:**
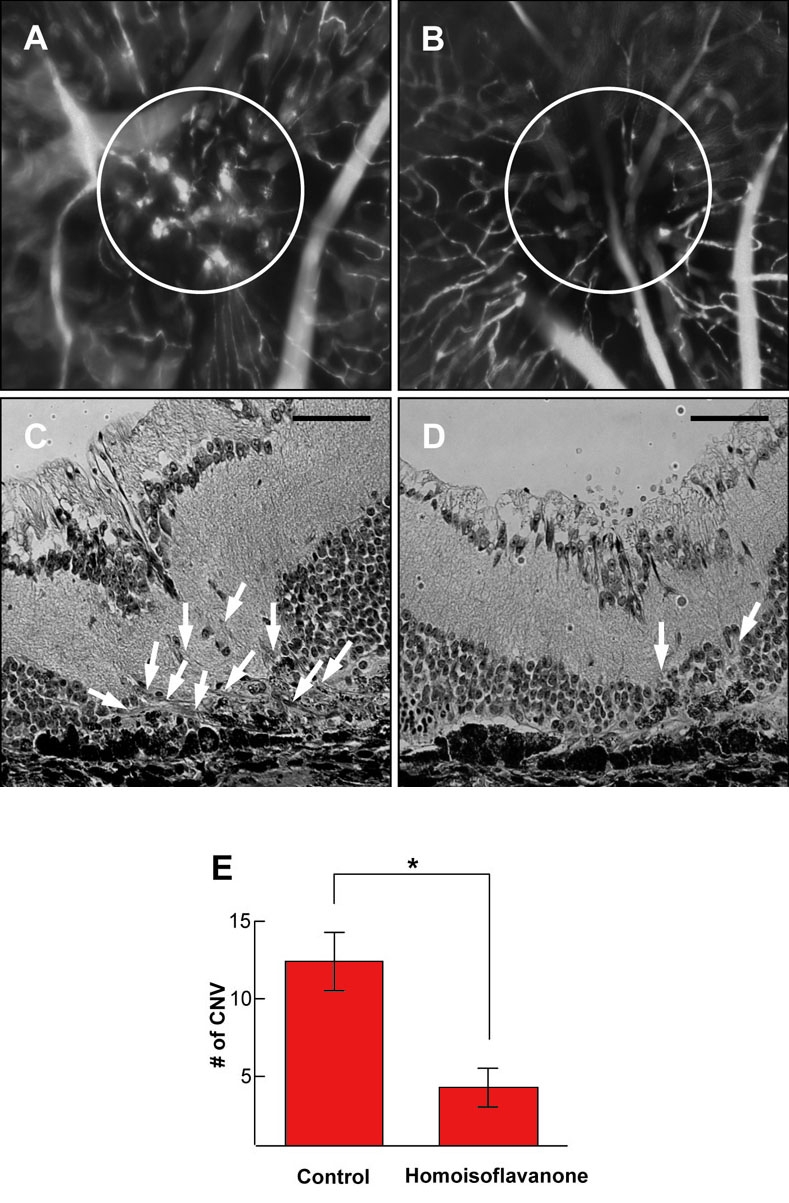
Effect of homoisoflavanone on laser-photocoagulation-induced choroidal neovascularization. Choroidal neovascularization (CNV) in control and homoisoflavanone-treated mice was evaluated by fluorescein angiography using 500,000 molecular weight fluorescein-conjugated dextran. Wholemount preparation from control (**A**) and 1 µM homoisoflavanone-treated (**B**) mice subjected to laser-photocoagulation-induced CNV was performed after 1 h perfusion of fluorescein-conjugated dextran, respectively. Circles indicate CNV in the laser-photocoagulation site. Hematoxylin-stained cross-sections were prepared from control (**C**) and 1 μM homoisoflavanone-treated (**D**) mice subjected to laser, respectively. Arrows show the new vessels growing from choroidal vessels. **E:**  To quantify CNV, we counted vessels from subretinal fibrovascular membrane. Data in each column are the mean ± standard deviation values from 100 sites of 25 mice (*p<0.05). Scale bars in **C** and **D** equal 50 µm.

### Qualitative assessment of choroidal neovascularization by fluorescein angiography

Fourteen days after laser photocoagulation we perfused the anesthetized mice with an intraperitoneal injection of ketamine-xylazine (10 mg/kg) through the tail vein with 500,000 molecular weight fluorescein conjugated dextran (Sigma–Aldrich Ltd., St. Louis, MO) dissolved in PBS. After 1 h perfusion the eyes of euthanized mice were enucleated and fixed in 4% paraformaldehyde for 4 h. The eyecups were dissected, flat-mounted in Dako mounting medium (DakoCytomation, Glostrup, Denmark), and viewed by fluorescence microscopy (BX50, Olympus, Tokyo, Japan) at a ×100 magnification.

### Quantitative assessment of choroidal neovascularization by counting vessels from subretinal fibrovascular membrane

Fourteen days after laser photocoagulation, the eyes were removed, fixed in 4% paraformaldehyde in 0.1 M phosphate buffer for 24 h, and embedded in paraffin. Sagittal sections of 4–5 μm, each 10 μm apart, were cut through the center of the laser-photocoagulation site. The sections were stained with hematoxylin and eosin to assess CNV via light microscopy (Carl Zeiss, Chester, VA). Any vessels from subretinal fibrovascular membrane were counted in five sections from each laser-photocoagulation site by two independent observers blinded to treatment (J.H.K. and K.H.P.). The average was calculated for over 100 sites of each group. There were at least 25 animals in each group.

**Figure 4:**
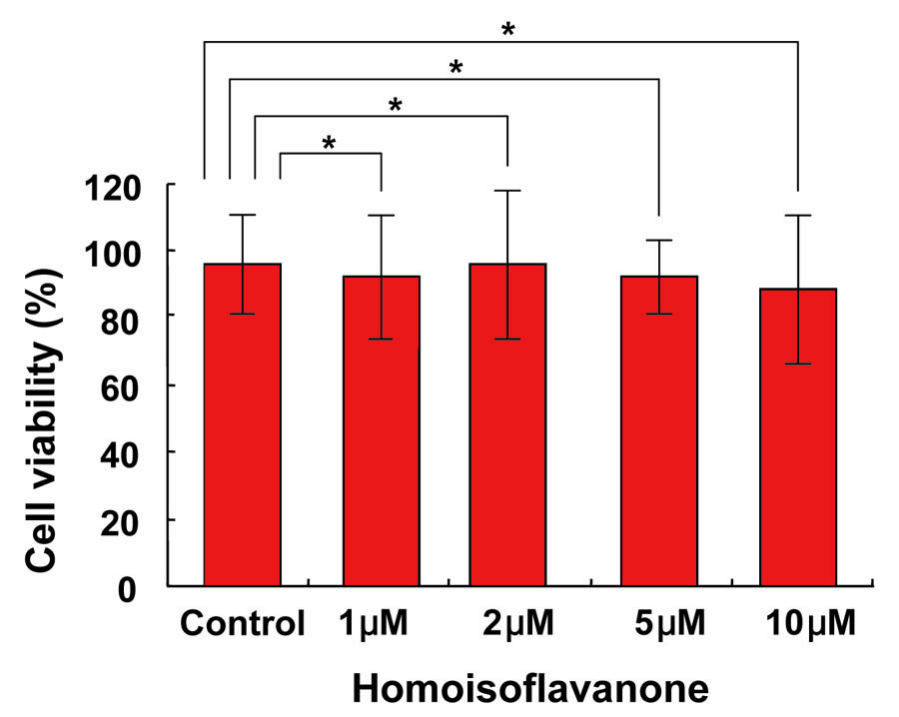
Effect of homoisoflavanone on the viability of human umbilical vein endothelial cells. Various concentrations of homoisoflavanone (1-10 μM) were treated on human umbilical vein endothelial cells (HUVECs), and cells were incubated for two days. Cell viability was measured by MTT assay. Each value represents means ± SEM from three independent experiments (*p>0.05).

### TUNEL assay

Ten μM homoisoflavanone was intravitreously injected into 7- to 8-week-old female C57BL/6J mice. For negative control, 1 λ PBS was injected. The mice of control or study group were sacrificed three days after 10 μM homoisoflavanone in 1 μl PBS injection or 1 μl PBS injection, and enucleated. Enucleated globes were fixed in 4% paraformaldehyde in 0.1 M phosphate buffer for 24 h and embedded in paraffin. Terminal deoxynucleotidyl transferase biotin-dUTP nick end labeling (TUNEL) staining was performed with a kit (ApopTag Fluorescein Green; Intergen, Purchase, NY), according to the manufacturer’s instructions. TUNEL-positive cells were evaluated in randomly selected fields at a ×400 magnification viewed under fluorescence microscopy (BX50, Olympus).

### Statistical analysis

Statistical differences between groups were evaluated with the Student’s unpaired t-test (two-tailed). Mean ± standard deviation was shown in figures. A p0.05 was considered significant.

## Results

### Effect of homoisoflavanone on tube formation of human umbilical vein endothelial cells

To investigate the effect of homoisoflavanone on an angiogenic phenotype of tube formation in vitro assay, we used FGF-2 as an angiogenic factor. FGF-2 induced the formation of extensive capillary-like networks of HUVECs cultured on two-dimensional Matrigel matrix. This effect was almost completely inhibited by co-treatment with homoisoflavanone (Figure 1A). FGF-2 stimulated tube formation of HUVECs approximately 1.4-fold, and this effect was abolished by homoisoflavanone (Figure 1B).

**Figure 5:**
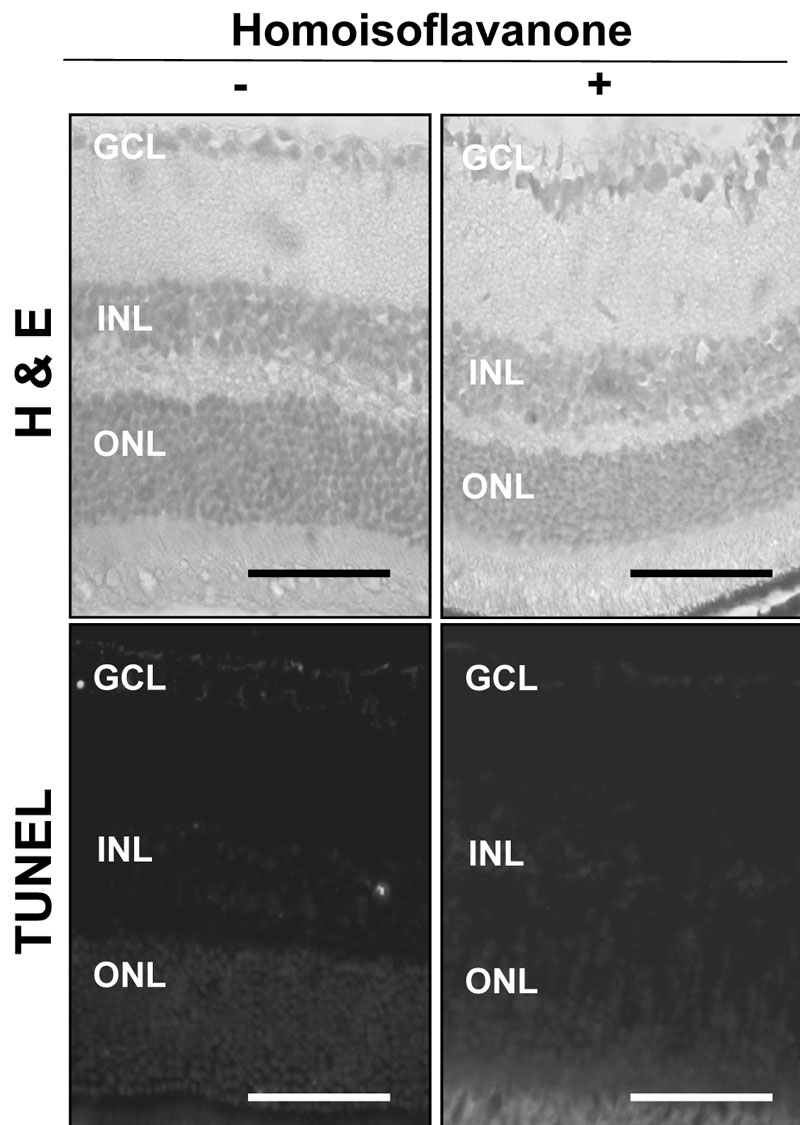
Retinal toxicity of homoisoflavanone. Ten μM homoisoflavanone was intravitreously injected, and the globes were enucleated three days after treatment. The retina was normal without any inflammatory cells in the vitreous, retina, or choroid. Compared to control, TUNEL-positive cells were not increased with homoisoflavanone injection. Abbreviations: ganglion cell layer (GCL); inner nuclear layer (INL); outer nuclear layer (ONL). Scale bars equal 50 µm.

### Effect of homoisoflavanone on cell migration of human umbilical vein endothelial cells

Consistent with inhibition of tube formation, FGF-2-induced HUVECs migration was blocked by homoisoflavanone (Figure 2A). FGF-2 increased chemotactic motility of HUVECs approximately 1.3-fold, which was nearly inhibited by homoisoflavanone (Figure 2B).

### Effect of homoisoflavanone on laser-photocoagulation-induced choroidal neovascularization

Based on our previous data of inhibition of in vivo angiogenesis of chick chorioallantoic membrane (CAM) assay and retinal angiogenesis without toxicity [7,8], we injected 1 μM homoisoflavanone in 1 μl PBS intravitreously, 10 days after laser photocoagulation when active neovascularization occurs. To assess CNV qualitatively, fluorescein angiography using fluorescein-conjugated dextran was performed. Retinas from control mice subjected to laser photocoagulation showed CNV with diffuse leakage on the laser photocoagulation site (Figure 3A). In contrast, retinas from homoisoflavanone-treated mice showed significantly reduced CNV and its leakage (Figure 3B). To quantify CNV, we counted, in a masked fashion, vessels from subretinal fibrovascular membrane. The vessels were defined as the mean number per section found in five sections per laser photocoagulation site. Retinas from control mice demonstrated multiple CNV patches (Figure 3C), whereas retinas from homoisoflavanone-treated mice showed significantly fewer neovascular lumens (Figure 3D). We found that homoisoflavanone-injected groups had a significant decrease of CNV compared to controls (Figure 3E).

### Effect of homoisoflavanone on the viability of human umbilical vein endothelial cells

To investigate cytotoxic effect of homoisoflavanone on HUVECs, we performed MTT assay in various concentrations of homoisoflavanone (1-10 μM). The viability of homoisoflavanone-treated HUVECs was not affected up to 10 μM (Figure 4).

### Retinal toxicity of homoisoflavanone

Retinal toxicity on intravitreal injection of 10 μM homoisoflavanone was evaluated through histological examination and TUNEL assay. As demonstrated in Figure 5, the retina was of normal thickness, and all retinal layers were clear without any inflammatory cells in the vitreous, retina, or choroid. Compared to control, TUNEL-positive cells were not increased with homoisoflavanone injection.

## Discussion

We first isolated homoisoflavanone from the bulb of *Cremastra appendiculata*, which has been traditionally used in Korea to prevent tumor metastasis, and found it to be an angiogenesis inhibitor through inhibitory activity against proliferation of HUVECs [7]. Previously, we demonstrated homoisoflavanone inhibits in vivo angiogenesis of chorioallantoic membrane of chick embryo [7] and retinal angiogenesis [8]. CNV and vascular leakage are two main causes of serious visual loss in AMD. We presented in this study significant inhibitory effect of homoisoflavanone on CNV. Homoisoflavanone reduced the incidence of clinically significant vascular leakage in an experimental model of CNV, which was consistent with histologic findings of a significantly lower number of CNV in homoisoflavanone-treated group.

The blood vessel growth in CNV correlates with the expression of vascular endothelial growth factor, FGF-2, and their receptors [10]. We showed that homoisoflavanone significantly inhibits FGF-2-induced tube formation and cell invasion of HUVECs. Previously, we revealed that G_2_/M phase cell cycle arrest, which is associated with the increase of p21^WAF1^ expression, caused the antiproliferative activity of homoisoflavanone on HUVECs [8]. In addition, homoisoflavanone showed no effect on cell viability of HUVECs, and no retinal toxicity up to 10 μM which is equivalent to 10 times of effective therapeutic dose (1 μM) to CNV. Based on these results, it is possible that homoisoflavanone may attenuate laser-photocoagulation-induced CNV through a direct antiangiogenic effect without cytotoxic effect in a therapeutic range (1-10 μM).

Given the well documented antiproliferative effect on HUVECs and antiangiogenic effect on and CNV in this study, homoisoflavanone, extracted from the bulb of Cremastra appendiculata, could be a new antiangiogenic agent for treatment of CNV. Furthermore, homoisoflavanone could be also applied to other vasoproliferative retinopathies, such as diabetic retinopathy, and tumors including retinoblastoma.
